# Effects of mechanical properties of polymer on ceramic-polymer composite thick films fabricated by aerosol deposition

**DOI:** 10.1186/1556-276X-7-261

**Published:** 2012-05-22

**Authors:** Oh-Yun Kwon, Hyun-Jun Na, Hyung-Jun Kim, Dong-Won Lee, Song-Min Nam

**Affiliations:** 1 Department of Electronic Materials Engineering, Kwangwoon University, 447-1, Wolgye-dong, Nowon-gu, Seoul, 139-701, South Korea

**Keywords:** Aerosol deposition, Al_2_O_3_, PMMA, PI, Integrated substrates, Composite films

## Abstract

Two types of ceramic-polymer composite thick films were deposited on Cu substrates by an aerosol deposition process, and their properties were investigated to fabricate optimized ceramic-based polymer composite thick films for application onto integrated substrates with the advantage of plasticity. When polymers with different mechanical properties, such as polyimide (PI) and poly(methyl methacrylate) (PMMA), are used as starting powders together with α-Al_2_O_3_ powder, two types of composite films are formed with different characteristics - surface morphologies, deposition rates, and crystallite size of α-Al_2_O_3_. Through the results of micro-Vickers hardness testing, it was confirmed that the mechanical properties of the polymer itself are associated with the performances of the ceramic-polymer composite films. To support and explain these results, the microstructures of the two types of polymer powders were observed after planetary milling and an additional modeling test was carried out. As a result, we could conclude that the PMMA powder is distorted by the impact of the Al_2_O_3_ powder, so that the resulting Al_2_O_3_-PMMA composite film had a very small amount of PMMA and a low deposition rate. In contrast, when using PI powder, the Al_2_O_3_-PI composite film had a high deposition rate due to the cracking of PI particles. Consequently, it was revealed that the mechanical properties of polymers have a considerable effect on the properties of the resulting ceramic-polymer composite thick films.

## **Background**

Due to the continuous demand for the miniaturization and integration of electronic devices for ubiquitous and digital convergence, system-on-package (SOP) technology is emerging as an alternative concept to overcome the technological limits of conventional technologies, such as system-on-chip and system-in-package. The SOP is a new concept for the three-dimensional integration of active and passive devices onto a single system to provide multifunctionality [1]. In order to integrate active and passive devices onto a single package for SOP, a fabrication technology for integrated substrates must be established. Ceramics are widely used for the integrated substrates since they have favorable characteristics, such as high reliability and good dielectric properties. Nevertheless, ceramics have a fundamental weakness in that they are easily fractured and require high-temperature processes for the fabrication of integrated substrates.

In the present technologies, two methods which overcome these fundamental weaknesses of ceramics for the fabrication of integrated substrates have been attempted. One is the development of a ceramic-polymer composite, which provides plasticity to the ceramics. The other is the use of low-temperature processes. However, during the formation of the ceramic-polymer composite, it was hard to increase the ceramic content above 60 vol.%. Even when the ceramic content in the composite was over 60 vol.%, the dielectric properties of the composites were worsened compared to those of pure ceramics. Furthermore, although low-temperature co-fired ceramics are well known in the low-temperature fabrication of ceramics, they still require high-temperature processes at around 850°C [2,3]. Taking these factors into consideration, the aerosol deposition (AD) process could be a good candidate method due to its various merits, such as room-temperature processing and the ability to form heterogeneous junctions consisting of different kinds of materials [4,5]. As reported in earlier studies, the AD process is based on shock-loading solidification via the impact of ultrafine particles with a surface. The consolidation and densification phenomena are affected by the mechanical properties of the starting powder [6,7]. To fabricate optimized ceramic-based polymer composite thick films by the AD process, it is necessary to understand how the mechanical properties of the starting powder affect the characteristics of the composite films. Thus, our focus was concentrated on the mechanical properties of the polymer powder. Poly(methyl methacrylate) (PMMA) and polyimide (PI) were chosen as the polymer powders for two reasons. First of all, PMMA and PI are widely used as matrix components in composite films due to their good electrical, physical, and mechanical properties [8-12]. However, these two polymers have different mechanical properties. It is reported that the tensile strength, fracture toughness, and elongation of PMMA are 48 to 76 MPa, 1.21 to 1.76 MPa·m^1/2^, and 2% to 10%, respectively. In comparison, the tensile strength, fracture toughness, and elongation of PI are 75 to 90 MPa, 1.65 to 5.4 MPa·m^1/2^, and 4% to 8%, respectively [13].

In this study, ceramic-polymer composite thick films were fabricated by the AD process and the effects of the mechanical properties of the polymer powder were examined for the two types of polymer. To confirm the effect of the mechanical properties of the polymer, Vickers hardness testing was carried out. Moreover, to understand how the mechanical properties affect the deposition characteristics, a high mechanical energy was applied to the two types of polymer powder using a planetary mill, and additional modeling was also carried out.

## **Methods**

The AD process involves the consolidation of ultrafine ceramic particles through impact with a substrate after the particles are accelerated using a carrier gas and a nozzle. Particles are mixed by means of a carrier gas and a vibration system in the aerosol chamber where they are aerosolized. Then, these particles in the aerosol state are accelerated through a nozzle by a pressure difference between the aerosol chamber and the deposition chamber. Then, thick and dense films are formed by impacting accelerated particles onto the substrate. The details of the AD process apparatus have been reported elsewhere [7,8]. Different ceramic-polymer composite films with various contents and types of polymer powder were fabricated on Cu substrates at room temperature by the AD process. The deposition parameters used for the AD process are summarized in Table 1. α-Al_2_O_3_ powder with an average diameter of 0.5 μm and a purity of 99.3% (A-161SG, Showa-Denko K.K., Minato-ku, Japan) was used as the ceramic starting powder. Two types of polymer powder with average diameters of 1.5 μm were used as the polymer starting powders: PMMA powder (MX-150, Soken Chemical & Engineering Co. Ltd, Toshima-ku, Japan) and PI powder (BMI-5100, Daiwa-Kasei Industry Co. Ltd., Okazaki, Japan). The microstructures and crystallinity of Al_2_O_3_-PMMA and Al_2_O_3_-PI composite films were examined by field-emission scanning electron microscopy (FE-SEM, S-4700, HITACHI Ltd., Chiyoda-ku, Japan) and X-ray diffraction (XRD, X’Pert PRO, PANalytical, Almelo, The Netherlands), respectively. Fourier transform infrared (FT-IR, Nicolet 6700, Thermo Fisher Scientific, Waltham, MA, USA) spectroscopy was employed to identify whether PMMA and PI exist in the deposited films. The Vickers hardness of the deposited films was measured using a hardness tester (TUKON 2100, Wilson Hardness, Norwood, MA, USA). The thicknesses of the coating layers were measured using a surface profilometer (XP-1, Ambios Technology, Santa Cruz, CA, USA).

**Table 1:** Deposition conditions of composite thick films fabricated by the AD process

**Deposition parameter**	**Value**
Consumption of carrier gas	1 to 10 L/min
Scanning speed of the nozzle motion	1 mm/s
Working pressure	5 to 20 Torr
Size of nozzle orifice	10 mm × 0.4 mm
Distance between substrate and nozzle	5 to 15 mm
Deposition temperature	Room temperature
Deposition time	10 to 30 min
Deposition area	10 mm × 20 mm

## **Results and discussions**

### **Fabrication of Al**_**2**_**O**_**3**_**-PMMA composite thick films**

**Figure 1:**
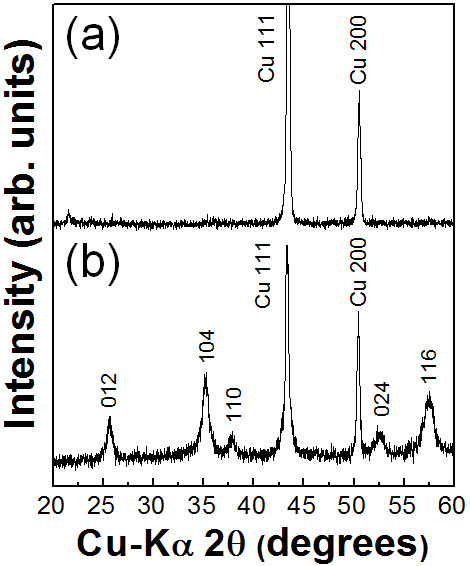
**XRD patterns of the (a) Al**_**2**_**O**_**3**_**-PMMA (30 vol.%) composite films and (b) Al**_**2**_**O**_**3**_**films.**

**Figure 2:**
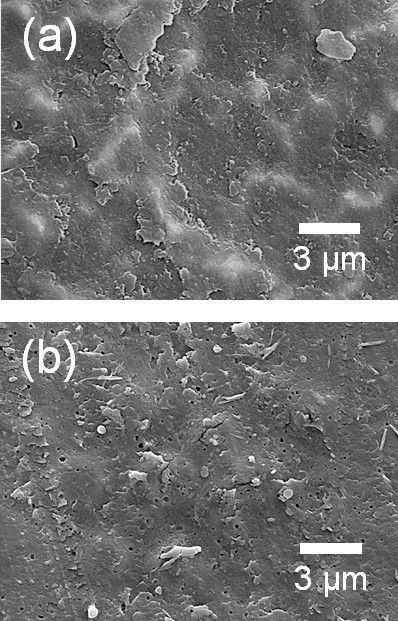
**Surface SEM images of the (a) Al**_**2**_**O**_**3**_**-PMMA (30 vol.%) composite films and (b) PMMA films.**

**Figure 3:**
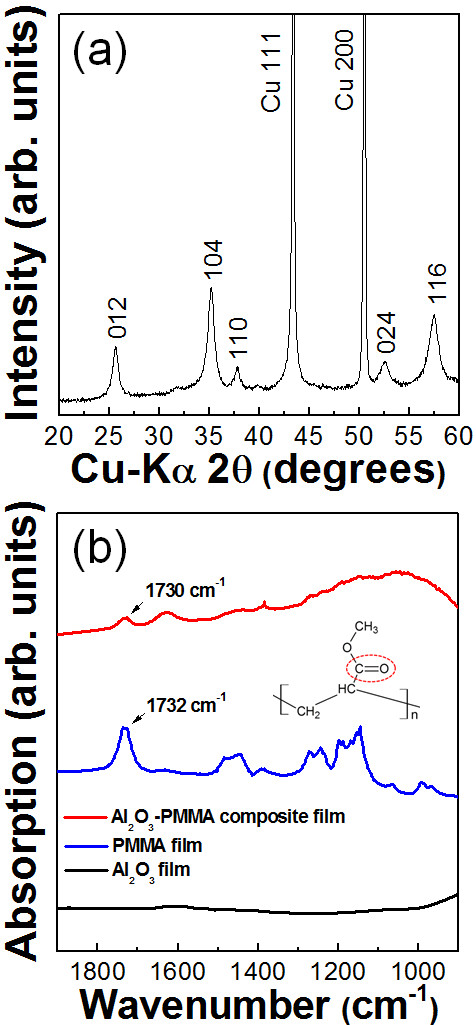
**XRD patterns and and FT-IR spectra.** (**a**) XRD patterns of the Al_2_O_3_-PMMA (15 vol.%) composite films. (**b**) FT-IR spectra of the Al_2_O_3_-PMMA (15 vol.%) composite, PMMA, and Al_2_O_3_ films.

**Figure 4:**
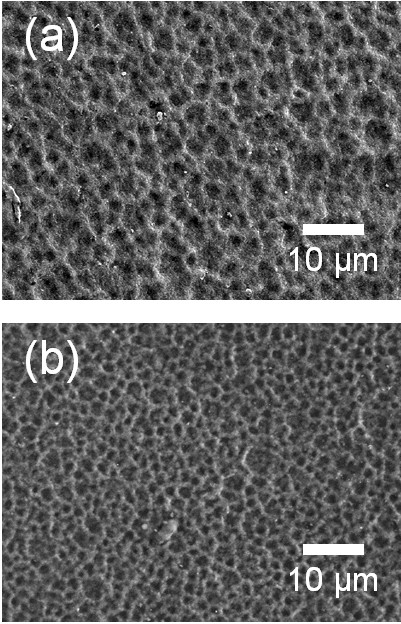
**Surface SEM images of the (a) Al**_**2**_**O**_**3**_**-PMMA (15 vol.%) composite films and (b) Al**_**2**_**O**_**3**_**films.**

Two types of polymer were used as the polymer starting powders in the fabrication of optimized ceramic-based polymer composite thick films by the AD process. First, the fabrication of Al_2_O_3_-PMMA composite thick films on Cu substrates was carried out. The content of the ceramic powder was chosen to be 70 vol.% because our goal was to fabricate ceramic-polymer composite thick films with ceramic contents of more than 60 vol.%. Therefore, the Al_2_O_3_ and PMMA powders were prepared with the initial volume ratio of 70 vol.% and 30 vol.%. The Al_2_O_3_-PMMA composite films were deposited on Cu substrates at room temperature by the AD process. To investigate the Al_2_O_3_ components of the deposited composite films, XRD analysis was carried out. As shown in Figure 1a, it was confirmed that there was no α-Al_2_O_3_ phase in the composite films deposited on Cu substrates, whereas the α-Al_2_O_3_ phase was confirmed in the XRD diffraction pattern of the Al_2_O_3_ films deposited on Cu substrates, as shown in Figure 1b. This means that almost all of the deposited films consisted of PMMA even though the contents of the Al_2_O_3_ starting powder were more than three times than the contents of the PMMA starting powder. We considered that these results were caused by the following factors: The first is the high density of the Al_2_O_3_ powder, which was three times higher than that of the PMMA powder. Thus, we expected that it was difficult to generate aerosol with Al_2_O_3_ powder than with PMMA powder. The second one is the low surface energy of the polymer. In general, polymer powders have low surface energy, which means that they are apt to push each other and disperse well in the aerosol, while ceramic powders tend to agglomerate due to their high surface energy. For these reasons, we thought that almost all of the deposited films would be consisted of PMMA since the concentration of PMMA powder in the aerosol was much higher than that of Al_2_O_3_ powder. In addition, SEM observation revealed that the Al_2_O_3_-PMMA composite films had similar surface morphologies to PMMA films, as shown in Figure 2a,b. Therefore, we thought that it is necessary to decrease the ratio of PMMA in the mixture. The volume percent of PMMA in the mixture was decreased from 30 to 15 vol.%, and further composite films were fabricated. For convenience, the Al_2_O_3_-PMMA mixture powder of 15 vol.% is referred to as the 15 vol.%-PMMA mixture. From the results of the XRD analysis, to investigate the α-Al_2_O_3_ components of deposited composite films, it was confirmed that α-Al_2_O_3_ phase existed in the deposited films, as shown in Figure 3a. In addition, FT-IR analysis was performed to verify the existence of PMMA in the composite films, as shown in Figure 3b. Although the presence of PMMA was confirmed in composite films, since the peaks at 1,730 cm^−1^ of the composite films were assigned to C = O stretching vibrations, it was considered that these PMMA components were present in small quantities because of the low intensity of the peak at 1,730 cm^−1^. Consequently, Al_2_O_3_-PMMA composite films could be fabricated by changing the content of the PMMA powder. However, the composite films contained very small amount of PMMA, and the deposition rate was also very low at 0.15 μm/min. To observe the surface morphology of composite films formed from the 15 vol.%-PMMA mixture, SEM analysis was performed. As a result, we found that the surface morphology of the films produced using the 15 vol.%-PMMA mixture, as shown in Figure 4a, has many craters, just like the surface of the Al_2_O_3_ film as shown in Figure 4b. From the above results, it was revealed that the surface morphology and components of Al_2_O_3_-PMMA composite films drastically depended on the amount of PMMA powder. This means that in the case of an Al_2_O_3_-PMMA mixture, it was hard to fabricate optimized ceramic-polymer composite thick films by the AD process.

### **Fabrication of Al**_**2**_**O**_**3**_**-PI composite thick films**

**Figure 5:**
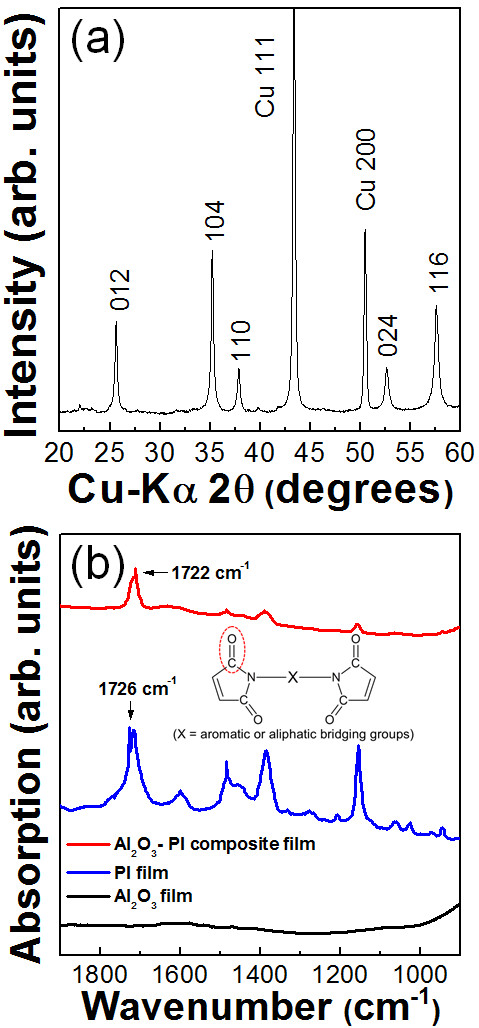
**XRD patterns and FT-IR spectra.** (**a**) XRD patterns of the Al_2_O_3_-PI (15 vol.%) composite films. (**b**) FT-IR spectra of the Al_2_O_3_-PI (15 vol.%) composite, PI, and Al_2_O_3_ films.

**Figure 6:**
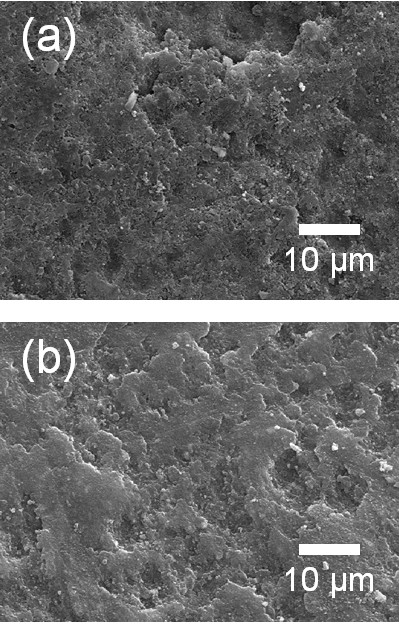
**Surface SEM images of the (a) Al**_**2**_**O**_**3**_**-PI (15 vol.%) composite films and (b) PI films.**

Al_2_O_3_-PI composite films produced using the 15 vol.%-PI mixture as a starting powder were deposited on Cu substrates at room temperature by the AD process. To investigate the Al_2_O_3_ components in the deposited films formed using the 15 vol.% PI mixture, XRD analysis was carried out. As a result, the presence of the α-Al_2_O_3_ phase in the composite films was confirmed, as shown in Figure 5a. FT-IR analysis was performed to verify the existence of PI in the composite films as shown in Figure 5b, and it was confirmed that PI exists in the composite films because the peaks at 1,722 cm^−1^ of the composite films were assigned to C = O stretching vibrations. The intensity of the peak at 1,730 cm^−1^ for the Al_2_O_3_-PI composite films was higher than that for the Al_2_O_3_-PMMA composite films. Therefore, it was considered that the polymer contents of the Al_2_O_3_-PI composite films were greater than those of the Al_2_O_3_-PMMA composite films. It is notable that the deposition rates were remarkably increased compared with those of the Al_2_O_3_-PMMA composite films. When using Al_2_O_3_-PMMA mixture as starting powder, the deposition rates of Al_2_O_3_-PMMA composite films were about 0.15 μm/min, as mentioned previously. In contrast, Al_2_O_3_-PI composite films showed much higher deposition rates of 1.5 μm/min. Also, the crystallite size of α-Al_2_O_3_ in the Al_2_O_3_-PI composite films was increased compared with that in the Al_2_O_3_-PMMA composite films. The crystallite size was calculated from the full width at half maximum of the diffraction peaks and Scherrer's formula. The crystallite sizes of α-Al_2_O_3_ in Al_2_O_3_-PMMA and Al_2_O_3_-PI composite thick films were about 35 ± 2 and 47 ± 2 nm, respectively. To observe the microstructures of Al_2_O_3_-PI composite films, SEM analysis was conducted. As a result, we observed that Al_2_O_3_-PI composite films had a similar surface morphology to that of PI films, as shown in Figure 6a,b, respectively. On the other hand, in the case of Al_2_O_3_-PMMA composite films, the surface morphology was similar to that of the Al_2_O_3_ films. In summary, the characteristics of ceramic-polymer composite thick films varied with the type of polymer powder used. This means that the type of polymer is an important factor in the fabrication of ceramic-polymer composite thick films by the AD process.

However, it has not been sufficiently clarified which properties of the polymer powder affect the characteristics of the deposited films. Therefore, as mentioned above, we focused on the mechanical properties of the polymer powder based on the mechanism of the AD process.

### **Comparison of Al**_**2**_**O**_**3**_**-PMMA and Al**_**2**_**O**_**3**_**-PMMA composite thick films**

**Figure 7:**
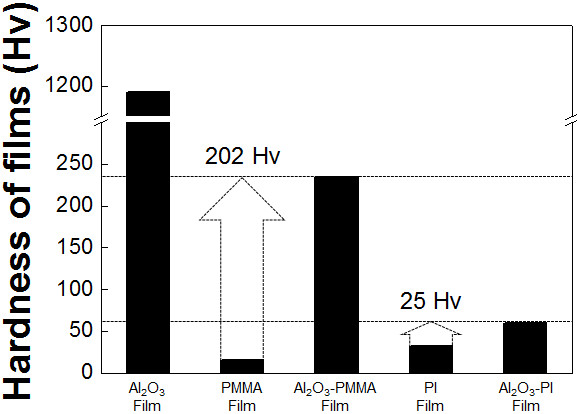
**The hardness results of PMMA, PI, Al**_**2**_**O**_**3**_**, Al**_**2**_**O**_**3**_**-PI, and Al**_**2**_**O**_**3**_**-PMMA films.**

**Figure 8:**
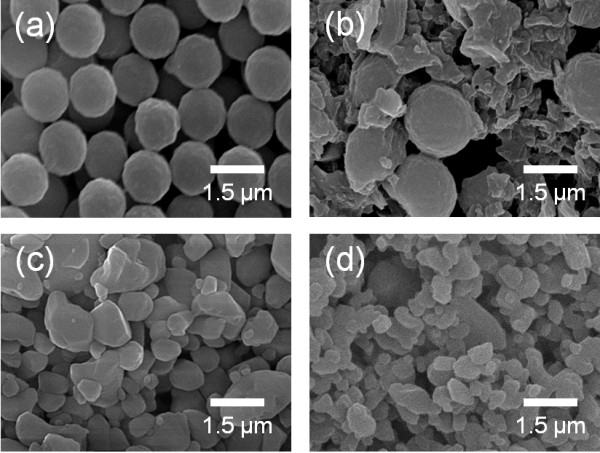
**Surface SEM images of the PMMA and PI powders before and after planetary milling.** Surface SEM images of the PMMA powder (**a**) before and (**b**) after planetary milling, and of the PI powder (**c**) before and (**d**) after planetary milling.

**Figure 9:**
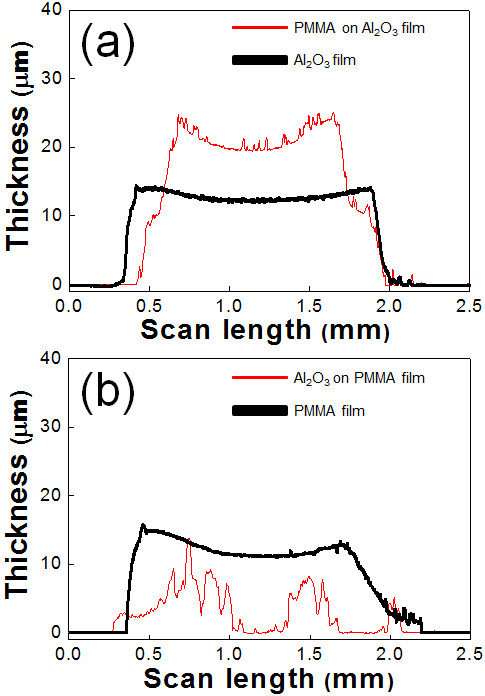
**The thickness of the (a) PMMA on Al**_**2**_**O**_**3**_**films and (b) Al**_**2**_**O**_**3**_**on PMMA films.**

**Figure 10:**
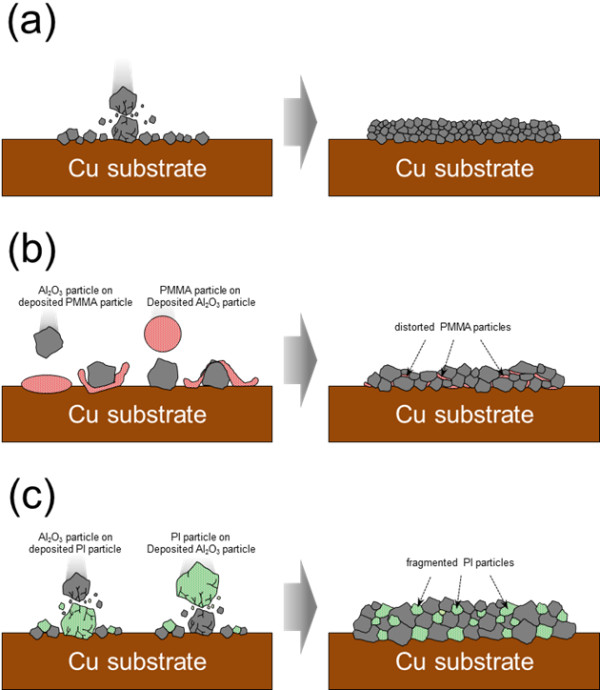
**Schematic diagrams.** (**a**) Al_2_O_3_ thick films being formed by the mechanical impacts of Al_2_O_3_ particles. (**b**) PMMA particles being distorted by Al_2_O_3_ particles.(**c**) PI particles being fragmented by Al_2_O_3_ particles.

To confirm the effect of the mechanical properties of the polymer, the hardnesses of PMMA, Al_2_O_3_, PI, Al_2_O_3_-PMMA composite, and Al_2_O_3_-PI composite films were individually determined by micro-Vickers hardness analyses. As shown in Figure 7, in the case of Al_2_O_3_-PMMA films, the hardness increased remarkably by about 2.1 GPa compared with that of PMMA films. In comparison, in the case of Al_2_O_3_-PI films, the hardness increased slightly by about 0.3 GPa compared with that of PI films. This means that the hardness of Al_2_O_3_-PMMA composite films was considerably affected by the hardness of Al_2_O_3_. On the other hand, the hardness of Al_2_O_3_-PI composite films was more strongly influenced by the hardness of PI than that of Al_2_O_3_. As a result, it was expected that the polymer contents of Al_2_O_3_-PMMA composite films were less than the polymer contents of Al_2_O_3_-PI composite films. Therefore, to investigate the cause of the difference in polymer content, a high mechanical energy was applied to the two types of polymer powder by planetary milling, since polymer particles impact the substrate and are impacted by Al_2_O_3_ particles during the deposition procedure. As a result, it was observed that the PMMA powder is severely distorted by the mechanical impact of a ceramic ball, as shown in Figure 8a,b. On the other hand, although the PI powder was fragmented into small pieces, the powder was not distorted, as shown in Figure 8c,d. Therefore, we can expect that the PMMA powder can be easily distorted by the mechanical impact of Al_2_O_3_ powder during the deposition procedure. Furthermore, in order to confirm the effect on the mechanical properties of PMMA by the impact of Al_2_O_3_ powder during the deposition procedure, we carried out modeling experiments as follows: At first, Al_2_O_3_ films and PMMA films were formed on glass substrates, and then depositions of PMMA and Al_2_O_3_ were conducted on the pre-deposited Al_2_O_3_ and PMMA films, respectively. After the attempted deposition of PMMA on Al_2_O_3_, it was confirmed that PMMA was deposited easily on the pre-deposited Al_2_O_3_ films using a surface profilometer, as shown in Figure 9a. On the other hand, Al_2_O_3_ was not deposited on the PMMA films, and pre-deposited PMMA films were etched by the impact of Al_2_O_3_ particles, as shown in Figure 9b. From the above results, it was confirmed that the mechanical properties of the polymer powder are one of the most important factors in promoting the fabrication of ceramic-polymer composite thick films by the AD process. From all the results achieved so far, we can explain the effect of the mechanical properties of the polymer powder. Generally, Al_2_O_3_ thick films deposited by the AD process were formed by the mechanical impact of Al_2_O_3_ particles as shown in Figure 10a. However, in the case of ceramic-polymer composite films, PMMA is distorted by the impact of Al_2_O_3_ particles during deposition and, eventually, the quantity of PMMA in the composite films is decreased, and the composite films had a low deposition rate as shown in Figure 10b. Although PI can be fragmented by the impact of Al_2_O_3_ particles, PI particles are deposited with Al_2_O_3_ particles as shown in Figure 10c. Consequently, we have revealed that the mechanical properties of the polymer are one of the dominant factors in promoting deposition during the AD process. The above results are expected to suggest guidelines for the fabrication of ceramic-polymer composite thick films by the AD process that can be applied to integrated substrates with the advantage of plasticity.

## **Conclusions**

Al_2_O_3_-PMMA and Al_2_O_3_-PI composite thick films were successfully fabricated on Cu substrates by the AD process; however, they had considerably different properties depending on the mechanical properties of their component polymers. The deposition rate and the crystallite size of α-Al_2_O_3_ in the Al_2_O_3_-PMMA composite film were decreased, and its surface morphology is similar to that of the Al_2_O_3_ film. In contrast, the Al_2_O_3_-PI composite film had a high deposition rate, its crystallite size was increased, and its surface morphology tended to be similar to that of the PI film. Through observations of the microstructures after planetary milling and additional modeling tests, it was revealed that the mechanical properties of polymers have a considerable effect on the properties of the ceramic-polymer composite thick films. In addition, we explained that the two types of polymer-ceramic composite films had different growth mechanisms: PMMA particles were distorted by the impact of Al_2_O_3_ particles during deposition, whereas PI particles were fragmented by the impact of Al_2_O_3_.

## **Competing interests**

The authors declare that they have no competing interests.

## **Authors’ contributions**

OYK, HJN, HJK, and DWL carried out the aerosol-deposited sample fabrication, measurements, and interpretation of the results. SMN initiated the idea of working on the present topic and analyzed all experiments. All authors read and approved the final manuscript.
